# Placenta Accreta Spectrum Disorders and Cesarean Scar Pregnancy Screening: Are we Asking the Right Questions?

**DOI:** 10.1055/s-0041-1731301

**Published:** 2021-06-28

**Authors:** Conrado Milani Coutinho, Laure Noel, Veronica Giorgione, Lígia Conceição Assef Marçal, Amar Bhide, Basky Thilaganathan

**Affiliations:** 1 Department of Gynecology and Obstetrics, Hospital das Clínicas, Faculdade de Medicina de Ribeirão Preto, Universidade de São Paulo, Ribeirão Preto, SP, Brazil; 2 Fetal Medicine Unit, St George's University Hospitals NHS Foundation Trust, Blackshaw Road, London, United Kingdom; 3 Vascular Biology Research Centre, Molecular and Clinical Sciences Research Institute, St George's University of London, London, United Kingdom

According to the World Health Organization, approximately 295,000 women died in 2017 during the antenatal and postpartum period. The vast majority (94%) of these cases occurred in low- and middle-income countries, with an estimate of 810 daily deaths from preventable causes. 1 Obstetric hemorrhage is the leading cause of maternal mortality worldwide and, among its key etiologies, placenta accreta spectrum (PAS) disorders have been increasing in prevalence concurrently with the global rise in the proportion of Cesarean deliveries and rates have currently being reported between 0.01% to 1.1% of pregnancies. 2 3 4 5 Accurate screening and diagnosis of PAS is of utmost importance for timely antenatal referral to tertiary hospitals and management by specialized multidisciplinary teams, which has been associated with a reduction in its associated morbimortality. 6 Although ultrasound diagnosis of PAS can be reliably done in centers with expertise, with an accuracy of approximately 90%, 7 8 in non-specialized facilities this rate falls to 50%, mainly due to insufficient clinical suspicion and/or knowledge of risk factors. 9 10 Therefore, effective and systematic screening and diagnostic protocols for PAS should be implemented in all maternal-fetal health care services in order to prevent adverse outcomes related to undiagnosed PAS disorders. The purpose of this article is to highlight the importance of basic questions that should be incorporated by all sonographers while performing routine obstetrical ultrasound to improve the detection of PAS.

## What are the Relevant Risk Factors for Pas Screening?

Numeroushistorical risk factors have been associated with the occurrence of PAS, including maternal obesity, advanced maternal age and parity, previous uterine surgery (including illegal terminations of pregnancy), and use of assisted reproductive technologies. 11 However, there is no doubt that the concomitance of the only risk factor related to the ongoing pregnancy–a low-lying placenta–with a previous Cesarean birth are the main risk factors for PAS, occurring concurrently in more than 90% of confirmed cases. 11 12 13 The reasons for that are not difficult to understand. Although preliminary studies suggested that PAS resulted from an excessive trophoblastic invasion and/or substandard decidual function, 14 15 the hypothesis of placental implantation on or into an iatrogenically defective decidua is currently gaining acceptance, 16 17 18 making the case for a common pathophysiological pathway between development of an uterine niche, Cesarean scar pregnancy (CSP) and PAS. Furthermore, recent epidemiological studies have challenged the previous association of the number of previous Cesarean sections and the risk for PAS, confirming that there is a plateau of risk for PAS after the second Cesarean birth. 19 20 This can be explained by the higher position of a uterine niche after previous elective Cesarean section compared to emergency Cesarean birth resulting in a three-fold increased risk of developing PAS in future pregnancies with placenta previa. 19 21 Therefore, as most risk factors for PAS seem to be proxy markers for the two previously cited and in order to improve the identification of PAS cases in the antenatal period, we would like to emphasize the importance of asking two simple questions while performing every obstetrical ultrasound: “is the placenta low-lying?” and “did the patient have a previous Cesarean section?”.

## Is First Trimester Ultrasound Screening for Paspossible?

**Fig. 1.**
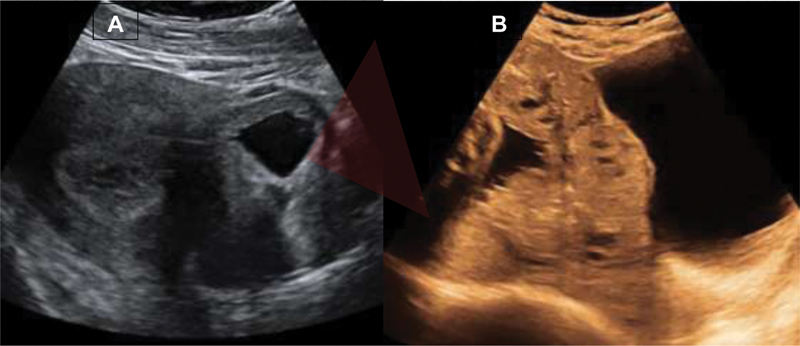
A. Sagittal first trimester transabdominal ultrasound image of a Cesarean scar pregnancy highlighting the “high-risk-for-placenta accreta spectrum triangle” (implantation on the lower anterior quarter of the uterus, and into the Cesarean scar niche); B. Sagittal third trimester transabdominal ultrasound image of a placenta accreta spectrum disorder on a placenta previa completely covering the cervical internal os (arrow).

If the answer is yes to the latter two questions, then it is indeed possible that the woman may have a PAS. As obstetrical ultrasound between 11 and 13 gestational weeks is conventionally performed for pregnancy dating, identification of multiple pregnancies, diagnosis of abnormalities and screening for trisomies and preeclampsia, this would be the perfect timing to firstly assess the risk for PAS disorders. Several ultrasound markers have been proposed, such as low implantation of gestational sac on or into a previous Cesarean scar, reduced myometrial thickness, placental lacunae, enhancedmyometrial vascularity and abnormal uterus-bladder interface, many of them in common with the diagnostic features of a CSP ( Figure 1 ). A 2018 systematic review and meta-analysis 22 concluded that at least one PAS sign can be identified during the first trimester in 91.4% of confirmed cases and that a low anterior implantation of the gestational sac or the placenta close to or within a previous Cesarean scar is the most commonly observed sign (82.4% of cases), with a sensitivity of 44.4% (95% CI, 21.5-69.2%) and a specificity of 93.4% (95% CI, 90.5%-95.7). In 2019, a prospective screening study 23 assessed the performance of a two-stage PAS screening strategy in 22,604 pregnancies. Patients were first evaluated between 11-13 weeks and those presenting low-lying placenta and a history of uterine surgery were referred to a specialized clinic at 12-16 weeks. For the 6% (1298 cases) of pregnant women with at least one marker and considered to be at high-risk, the diagnosis of PAS was suspected in 14 cases and confirmed in 13. There were no cases of PAS in the low-risk patients. Performance of screening was not assessed due to the low number of PAS cases. These findings support the relevance of being aware of the position of the gestational sac/placenta in the first trimester scan in patients with a history of Cesarean sections, especially for the high-positioned scars secondary to elective procedures. On the one hand, the first trimester diagnosis of a CSP/PAS is desirable and should be pursued, mainly for being a condition associated with increased maternal morbimortality with a need for referral to specialized multidisciplinary centers for appropriate counselling and management. 24 On the other, this first trimester screening strategy would label 6% of women as being at high-risk for PAS, resulting in additional expenditure, use of human and logistical resources, and the negative psychological burden on the family – with less than 1 in 100 of these 'high-risk' women actually having a PAS. Additionally, although termination of pregnancy is usually discussed with these families, the natural history of CSP is not yet fully understood. Recent studies tried to discriminate the outcomes of CSP based on ultrasound signs. Among them, placental implantation “in the niche” instead of “on the scar”, 25 residual myometrial thickness below 2 mm, 25 and identification of the pregnancy in the “high-risk-for-PAS triangle”, 26 would be predictive of worse surgical outcomes and more advanced third-trimester sonographic staging of PAS ( Figure 1A ). However, the rarity of this condition precludes the assessment of strong associations with outcome from the previous studies. Therefore, it is imperative to establish a collaborative approach to gather global experience among specialists conducting CSP cases. With this purpose in mind, we encourage clinician to upload CSP cases onto the international CSP Registry ( https://csp-registry.com ) ( Figure 1B ).

## Contingent Second and Third Trimester Screening for Pas

**Fig. 2.**
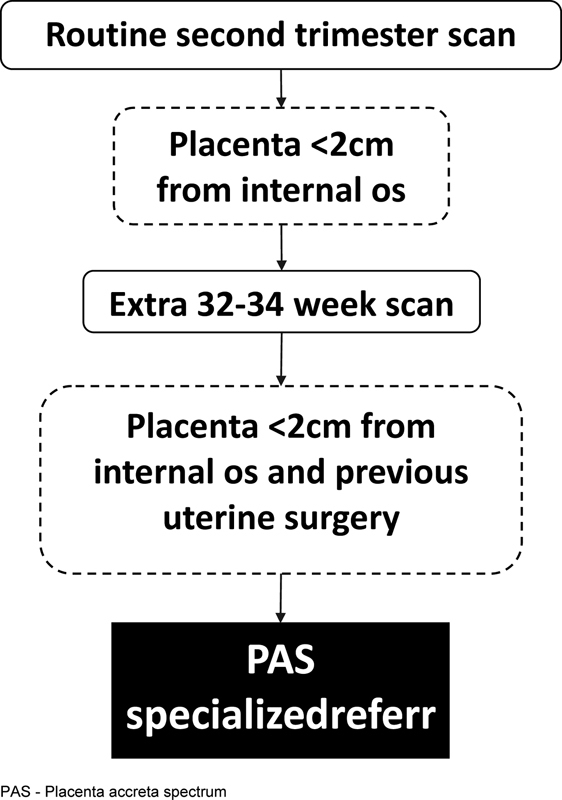
Flowchart illustrating the screening pathway for PAS starting from the mid-trimester and highlighting the importance of the implementation of two simple questions on routine scanning: (1) “is the placenta low-lying?” and (2) “did the patient have a previous cesarean section?”

The rationale for a mid-trimester screening for PAS is to take advantage of the conventional 18-23 weeks anatomical ultrasound evaluation and the already implemented screening for placenta previa in non-specialized facilities. With the two proposed questions in mind, upon identification of a low-lying placenta (first question) on routine mid-trimester scan, all sonographers should enquire the patient about a previous Cesarean section (second question). The order of these questions is extremely important for the feasibility of the screening program, as the proportion of patients with previous uterine surgery is incomparably higher than those with persistent low-lying placenta in the third trimester. This strategy has been explored by a retrospective study encompassing 57,179 women scanned between 18-23 gestational weeks. 27 For the 7.8% of patients with a low-lying placenta, a 32 week scan was arranged to assess placental position. Only 220 (0.4%) had a diagnosis of persistent placenta previa. 75 (0.1%) of them had a previous uterine surgery and were therefore referred for assessment by the PAS diagnostic service. In total, 21 out of 22 PAS cases were correctly identified by this screening program, with a sensitivity of 95.45% (95% CI, 77.16-99.88%) and a specificity of 100% (95% CI, 99.07-100%) ( Figure 2 ). PAS was confirmed based on clinical and histopathological criteria, as recommended by the International Federation of Gynecology and Obstetrics (FIGO). 28 From a public health perspective, this contingent PAS screening strategy is feasible in lower-resource medical settings with basic obstetric ultrasound facilities, not requiring additional visits beyond those that are routinely indicated. Furthermore, comparing to the first trimester screening, only 0.1% of patients would need to be referred to a specialized PAS diagnostic service (with one in three having a confirmed PAS), as opposed to a 6% figure between 11-13 weekswith less than one in 100 subsequently diagnosed with PAS. The success of such a screening strategy relies on an established regional referral service, with access to fetal medicine specialists properly trained to diagnose PAS disorders and dedicated, highly specialized multidisciplinary team at tertiary level hospital, where safe delivery can be arranged. 29

Two simple questions asked by the sonographer at every obstetric ultrasound examination have the potential to alter the course of pregnancies at risk for PAS: (1) “is the placenta low-lying?” and (2) “did the patient have a previous cesarean section?”. Suspicionfor CSP during the first trimester scan should trigger referral to specialized centers and careful counselling taking into consideration the lack of data regarding the natural history of CSP. Contingent screening for PAS in women with persistent placenta previa in the third trimester and a history of previous Cesarean section is feasible, effective and does not put additional burden on the public health system. In parallel with the establishment of specialist referral centers, the implementation of these simple questions and screening strategy have the potential to improve antenatal PAS detection rates and decrease maternal morbidity and mortality secondary to undiagnosed PAS.
